# Are Dentists' Exempt from Jury Duty?

**Published:** 1883-04

**Authors:** 


THE
AMERICAN JOURNAL
OF
DENTAL SCIENCE.

Vol. XVI. THIRD SERIES—APRIL, 1883. No. 12.

### ARTICLE I.

### *Are Dentists' Exempt from Jury Duty?*

THE FOLLOWING CASE IS OF INTEREST TO THE PROFESSION.

*Letter of Counsel*, WASHINGTON, D. C., *February* 10*th*, 1883.

Dr. ——

DEAR SIR: As a counsel for the Washington Dental Society, we are requested to communicate with you as a member of said society, and to give you the result of our research and investigation as to the question whether a dental surgeon, duly licensed as such by a college having the requisite faculties, or by a university, and practicing his profession in the District of Columbia, is exempt from jury duty in this district. You may remember that the late lamented Judge Olin held, in the case of Dr. ——, a dental surgeon summoned as a juror, that he was exempt from jury duty. The case is not reported, but you can find from Dr. —— the occasion of the decision and the reasons given by Judge Olin. Our act of Congress on the subject of exemptions, and the only act of Congress on the subject, was passed June 16th, 1862, just about nine months before the Supreme Court of the District of Columbia was organized. It is incorporated in the Revised Statutes of the

United States relating to the District of Columbia, section 875. It is affirmative legislation, and may not have the effect of repealing prior laws making exemptions not therein mentioned, as in the case of The King vs. Pugh, 1 Doughlas, 191, where Lord Mansfield, delivering the opinion of the court, said: "We have considered this matter very fully, and we are all of opinion that the statutes relative to juries' being affirmative, do not take away the prior exemption." The only Maryland acts of assembly touching exemptions from jury duty, which were passed prior to February, 1801, when Congress passed the act adopting the laws of Maryland then in force, were the acts of 1715, ch. 37, § 4, and 1797, ch. 87, § 6, neither of which related to physicians or surgeons. It is remarked by Chancellor Kilty, of Maryland, in his report to the Maryland Legislature on the British Statutes, in force in the provinces, page 230, that the statute of 5 Hen. 8, chap. 6 (1513,) although in its terms applicable only to surgeons of London, is supposed, together with others respecting physicians, to have extended to the kingdom generally." It is stated in 3 Bl. Comm., 364, that the exemption from serving on juries is extended by divers statutes, customs, and charters, to physicians and other medical persons; and Kilty says: "It has not been the practice to summon them on juries in the province or in the state, although they are not exempted by the act of 1715, ch, 37, from which it may be inferred, that these statutes extended to the provinces." Chancellor Kilty was the Chief Justice of the Circuit Court of the District of Columbia. His report on the British Statutes, existing at the time of the first emigration and by experience found applicable, was printed and distributed under the sanction of the state for the use of its officers, and is a safe guide, says Judge Buchanan, delivering the opinion of the court in Dashiel vs. Attorney-General, 5 Harris & Johnson, p. 403; and Alexander, in his Collection of British Statutes in force in Maryland, p. 277, says, that surgeons, physicians, and other medical persons are not even exempted from jury service by the Maryland Code, but that they are exempt, as it is presumed, by force of these early statutes. Alexander gives in full the text of act of 5 Henry VIII., cap. VI., as being still in force in Maryland, and thereby surgeons are exempt from jury duty and also barber surgeons. Letters-patent were granted in first year of Edward IV. to the freemen of the mystery of barbers using the mystery or faculty of surgery, by which they become a corporate body. In the 32d year of Henry VIII., as there were two distinct companies in London "occupying and exercising the science and faculty of surgery," the one company being called "The Barbers of London" and being incorporated, and the other company being called "The Surgeons of London" and not being incorporated, both companies were united and made one body corporate by act of Parliament, 32 Henry VIII, cap. 40, and called by the name of "Masters or Governors of the Mystery and Commonalty of Barbers and Surgeons of London," and they were made exempt from inquest duty. It was enacted by the same act, that "no manner of person within the City of London, suburbs of the same, and one mile compass of the said City of London, * * * shall occupy any surgery, letting of blood, or any other thing belonging to surgery; drawing of teeth only except." And surgeons within the same limits were forbidden to "occupy" or "exercise the feat or craft of barbery or shaving." The said corporation of barbers and surgeons continued until 18 George II., when the surgeons and barbers were made two separate and distinct corporations, reserving the privileges each were entitled to under Stat, 32 Henry VIII., to each company severally, vide Stat. 18 George II., cap. XV.

The barbers continued to have the same privileges after 18 Geo. II., as before, "so far as the same do not concern or relate to the art and science of surgery," and "with respect to everything but surgery." The surgeons so continued to be incorporated till 1800, when the Royal College of Surgeons was established. As the statute of Henry

VIII. allowed the barbers to continue in one part of surgery, the drawing of teeth, and prohibited them from practising every other part of surgery, even treating the diseases of the teeth, the barber, who was a mere tooth-drawer, so far as surgery is concerned, after the act of Henry 8, chap. 40, was not, it would seem, allowed to practice this part of surgery after the act of George II. for reward or compensation. Thereafter dentistry arose and has made such wonderful strides that parliament, in 1859, recognized them as surgeons, and granted authority to the Royal College of Surgeons to give diplomas in Dental Surgery. Dentists were not barbers, as has been vulgarly supposed, although barbers were at one time surgeons. The dentist was a surgeon from the start and after the surgeon had ceased to be a barber. He draws teeth, it is true, but he draws them very seldom, and only when absolutely necessary. He is not a destroyer or extirpator of teeth. He is a preserver of them, so intimately acquainted with their structure and organism that he can replant them when necessary, and even transplant teeth from one person to another. He understands all their diseases and the treatment of them, and all the diseases of the oral cavity and their treatment.

The barber, whilst a mere tooth-drawer in surgery, was exempt from jury duty. The surgeon was, also, exempt as we have above seen. The policy of the exemption was on account of the inconveniences and possible hurt or even danger to the public by reason of the absence from their professional duties of persons specially skilled in any part of the healing art. The act of George II. exempted surgeons, after their separation from the barbers, from all "parish, ward, and leet offices, and of and from the being put into or serving upon any jury or inquest," and provides that any surgeon summoned and returned to serve on a jury "shall be absolutely discharged from the same," and the return "shall be utterly void and of no effect."

The act of 6 George IV., c. 50, § 2, specifies the persons exempted from jury duty and declares that their names shall not be put upon the jury lists, and, among others, "all surgeons being members of one of the royal colleges of surgeons in London, Edinburgh, or Dublin and actually practicing." Now, it was an inconvenience to the public and a hardship on the dental surgeon that he should not be exempt from jury duty when he was recognized by Parliament and by the Royal College of Surgeons as a surgeon, simply because he might not be a member of one of the royal colleges of London, Edinburgh, or Dublin. This patent incongruity in the law was corrected by Parliament by an act passed July 22d, entitled "An act to amend the law relating to dental practitioners." It provides first for the registry of persons qualified to pursue the profession of dentistry, and then enacts as follows: "Every person registered under this act shall be exempt, if he so desires, from serving on all juries and inquests whatsoever, and from serving all corporate, parochial, ward, hundred, and township offices, and from serving in the militia."—(Law Reports, Stat. 13, page 269.) This act clearly shows that dental surgeons are within the classes of persons which it is within the policy of the law to exempt. At the annual meeting of the Metropolitan Counties' Branch of the British Medical Association, held at Crystal Palace in July, 1881, Edwin Sanders was elected president in place of Sir Henry Thompson, and in the course of his address said: "Concurrently with a rapid and brilliant advance in the science and art of dentistry, due to a large extent to a wave of progress which reached this country from America, there arose an increasing demand for and an appreciation of its services on the part of the public." He was speaking of a time before the year 1860, and then speaking of the want felt after that time for a registry as a check to the intrusion of the unprincipled and uninstructed, he says; "This has now been happily accomplished by the dentists act of 1878, and thus legislative sanction has been obtained for a scheme not directed to give prominence to the educated and qualified few, but to raise the whole body of the profession; not to accentuate the distinctive character of the specialty, but indissolubly to unite it to the great surgical body through the examining board of the Royal College of Surgeons." Dental surgery has a section in the International Medical Congress. It has, also, a section in the American Medical Association. The universities of *Maryland*, Michigan, Pennsylvania, Iowa, Tennessee, California, Vanderbilt, Howard, and Harvard have organized in connection with their medical departments, as you well know, departments known as dental departments, and confer the degress of D. D. S. We know that in England before 1862, the date of the passage of the act of Congress, the dental surgeon was treated as a surgeon and dental surgery as a branch of surgical practice, and this was due, said Professor Sanders, to "a wave from America." In this country, therefore, prior to 1859, when the act of Parliament was passed giving the Royal College of Surgeons power to grant diplomas in the specialty of dental surgery, the dental surgeon was recognized as one having not only a professional standing, but as having a specialty duly recognized as a part of medical science. In this condition of things the act of Congress of June 16th, 1862, was passed, and in 1874, § 875 of the Revised Statutes D. C. was enacted, whereby it is provided that all practicing physicians and surgeons shall be exempt from jury duty. Can it be said that a dental surgeon in 1862 or in 1874 was not fully recognized in this country as well as in England as a surgeon ?

The act says all surgeons shall be exempt. Who is a surgeon ? What is a surgeon ?

There is a current idea that a surgeon is one who cuts and carves the human frame, and hence the words scalpel of the surgeon are so often found in alliterative company, like the words palette of the painter, sword of the soldier, or brief of the barrister. The word surgeon does not necessarily imply the idea of cutting, for, if so, the lithotritist would not be a surgeon. The word *surgeon* is derived from the old French *surgien*, which is a contradiction or rather corruption *chirurgien*, (the chi being pronounced she in this word in French) which is made up of two Greek roots signifying " hand " and " work," and is applied to one who practices " that part of the healing art which relates to external diseases and their treatment, especially to the manual operations adopted for their cure." (Worcester.) See, also, to the same effect, Webster, Jacob's, Tomlin's Law Dictionary, Dunglison's Medical Dictionary and Cooper's Dictionary. The lithotritist, although he may be a surgeon of greatest skill, is not likely to have the same skill for the diseases of the ear or eye, of the teeth or throat, as the aurist or oculist, as the dentist or laryngosopist, and is not the less a surgeon because he refers to the laryngoscopist all cases where the throat is diseased, to the dentist, where the teeth or oral cavity are diseased, to the aurist where the ears or contiguous parts are diseased, and to the oculist where the eyes are diseased. If the aurist be a surgeon, and he is so described in the dictionaries, is he less a surgeen because he lets the lithotritist grind a stone or the embryotomist crush a fœtus? If the oculist be a surgeon, and he is so described in the dictionaries, is he less a surgeon because he lets an ovarian tumor be removed by a specialist in that line? If the laryngoscopist be a surgeon, and his cunning is so new that he is not as such mentioned in the dictionaries, is he less a surgeon than his older brother the dentist, who is old enough to be described in the dictionaries, and who is described therein as a surgeon, because the surgery of the teeth and adjacent parts are by him, the laryngoscopist and all the other surgeons, confided to the dentist?

We think the dictionaries well describe aurist, dentists, and oculists to be surgeons. We look at Worcester and we find: Aurist, " a surgeon who treats diseases of the ear ;" dentist, " one who devotes himself to the study of the diseases of the teeth and their treatment ; a surgeon of the teeth, called also dental surgeon and surgeon dentist ;" oculist, " a surgeon who occupies himself chiefly with the management of diseases of the eye."

We think that when the act of Congress says all surgeons shall be exempt, it cannot be successfully maintained that some surgeons are not exempt, and especially that some such surgeons as it is the policy of legislation to exempt, are not exempted. It would have been wiser to particularize the surgeons to be exempted, and select only such surgeons as have been licensed by some standard college or university, as Parliament has done; but we are dealing with the act as it is, and not as it ought to be. We think that you are exempt, and that His Honor Mr. Justice MacArthur will permit you to test the question in the court in general term. Assert your right to exemption by challenge and motion, supported by affidavit.

Yours truly,

APPLEBY & EDMONSTON.

---

IN THE

*Supreme Court of the District of Columbia*

---

*In re* Dr. —— } Circuit Court, February, 1883.

MOTION.

Dr. —— now comes into this court, this thirteenth day of February, A. D. 1883, and challenges himself as a juror, and moves the court to discharge him from jury duty, on the ground that by the law he is exempt, for the reason that he is a practicing surgeon in the District of Columbia, as shown by the affidavit filed herein, which he tenders himself ready to verify.

Dr. ——.

Witness:

GEO. F. APPLEBY.

ORDER OF COURT.

In the matter of Dr. ——, praying a discharge from jury duty, because he is a dental surgeon, practicing as such, the motion and affidavit in support thereof are hereby respectfully referred to the Court in General Term, there to be heard in the first instance.

MACARTHUR, *Justice.*

---

AFFIDAVIT OF DR. ——.

This affiant, having been duly sworn according to law, states that he is a resident of the District of Columbia, a citizen of the United States, over twenty-one years of age, and has never been convicted of any felony or misdemeanor whatever-

This affiant further states, that for more than twenty-five years he has practiced the profession of a dental surgeon and still practices; that he was licensed to practice said profession by a College of Dental Surgery, chartered by an act of the Legislature of the State from which college he was graduated, having pursued the full course of study here prescribed. That he has a diploma of said college admitting him to the degree (chirurgiæ dentium doctoris) of a Doctor of Dental Surgery (D. D. S.) That the *curriculum* of study in said college embraces pathology and therapeutics, anatomy and physiology, chemistry and materia medica, oral surgery and metallurgy, as well as operative and mechanical dentistry. That by said studies a knowledge of the pathological relations of the teeth to the other parts of the system is obtained, together with the symptoms, causes and treatment of all diseases which involve the dental structure, such as inflammatory action affecting the various tissues, diseases of the dental pulp, periodontitis, alveolar abscess, dental exostosis, dental caries, necrosis, &c., &c.; that thereby knowledge, is obtained to ensure careful attention to the chemistry of metals, and to the vital chemistry of anæsthetics; that thereby knowledge is obtained so as to construct and apply instruments and appliances for correcting irregularities of the teeth or dental arch, treatment of dislocation and fractures of the maxillæ, removal of morbid growths, treatment of ulceration of the tongue and any disesse of the antrum. That the text-books used in said college and in kindred colleges are Garretson's Oral Surgery, Kinsley's Oral Deformities, Harris' Principles and Practice of Dental Surgery, Richardson's Mechanical Dentistry, Gross's System of Surgery, Paget's Surgical Pathology, Miller's Principles of Surgery, Gray's, Wilson's or Handy's Anatomy, Towme's Dental Anatomy, Dalton's, Flint's, Draper's, or Kuss' Physiology, Fowne's, Rosco's or Wilson's Chemistry, Bowman's Practical Chemistry, Wood's Therapeutics, Biddle's Materia Medica, Wedl's Dental Pathology, Beal on the Microscope, and other works. That for said studies, illustrations are made, both upon the living subject and the cadaver, by dissections of the cadaver, and by diagrams, preparations and models.

This affiant further shows, that he has acquired a large practice in the District of Columbia, and has his time during the hours of the day constantly employed by the very exacting demand of his patients, and sometimes the hours of the night. His patients are oftentimes sufferers from excruciating pain, and require immediate surgical treatment for relief. He has constantly on hand what are called "regulating cases." In these it is necessary for him to see at regular intervals the child or young person whose crooked teeth are being regulated or straightened; in some of these cases daily, and in others two, three, or four times a week. The springs or ligatures which move the teeth towards the position they are designed to occupy, require constant adjusting, and if they are not regularly attended to the patient suffers materially; and if, by neglect to remove them, the enamel should be softened, great and permanent injury may result. The movement of the teeth having been begun, should go on regularly and uninterruptedly, for if the fixtures are not attended to and altered frequently, so as to keep up the requisite pressure, the teeth will in a short time become more or less fixed and tightened where they are, and renewed movement after a time is not only more difficult, but causes much additional pain and distress to the little patient, the most painful part of the operation being the starting of the movements in the teeth. These cases each range in treatment from one to twelve months.

This affiant further states, that cases are constantly occurring in his practice where arsenic has to be used to devitalize a pulp in order to preserve a tooth. In such cases he is required to see the patient at a certain time after the application is made, in order to remove the arsenic and extirpate the dead pulp. If the arsenic be not removed in due time, the tooth will become discolored and an abscess will most probably result, giving rise not only to great pain and discomfort, but to serious and possibly permanent injury and disfigurement, by resulting necrosis of a portion of the alveoli, or even endangering life through the absorption of pus into the system, causing pyæmia.

This affiant further states, that, being in full practice, he is constantly consulted by patients for the treatment of abscesses occurring at the roots of "dead" teeth, of necrosis of the alveoli or any portion of the maxillæ, for treatment of the disease of the antrum, of ulceration of the tongue, of tumors of the gums, of fractures of the jaw and teeth, and in cases of interrupted second dentition. In the last named cases a reflex constitutional disturbance is oftentimes caused by interrupted second dentition, which is overlooked by the medical attendant, and serious nervous derangements have resulted to the young subjects, which have been rapidly cured by the application of local surgical treatment.

This affiant further states, that fractures of the jaw, though not occurring so frequently as fractures of other bones of the body, are liable to be met with at any time, and, when occurring, require prompt, skillful, and continued treatment ; and these cases properly belong to the dental surgeon, who has succeeded in their treatment and cure when the resources of the general surgeon have signally failed, as in the well-known case of the Hon. Wm. H. Seward, who was injured in Washington City whilst Secretary of State.

This affiant further states, that teeth, and especially front ones, are not infrequently loosened, and are sometimes entirely knocked out, by accident. The timely aid and special skill of the dental surgeon can replant the teeth when knocked out and render them permanently firm and useful.

This affiant further states, that hemorrhages, resulting from the particular diathesis of the patient and from other causes, frequently follow the extraction of teeth, sometimes immediately and sometimes after the lapse of hours and days. Secondary hemorrhage, when occurring, is known by physicians and surgeons to be more dangerous than primary, and these cases are always treated by the dental surgeon, as he by his special education and study of the detailed anatomy of these parts, is better qualified for their treatment, and when such cases come to the notice of the physician they are generally referred to the dental surgeon. And this affiant further states, that he has constantly on hand, and there is not a dental surgeon of full practice who has not, cases which require continued and frequent treatment and applications of medicaments for days and weeks, governed by the diathesis and constitutional condition of the patients.

This affiant further states, that he has books and pamphlets in his possession or control by which, it will appear to the court, that his profession is regarded as an important collateral branch of medical science. That the Universities of Maryland, Harvard, Pennsylvania, Michigan, Iowa, Tennessee, Howard, Vanderbilt, and California have instituted and organized, in connection with their medical departments, departments that are known as Dental Departments, which are now in active operation, and confer the degrees of D. D. S, ; that the American Medical Association has established a section of dental surgery; that on September 8th, 1859,, by act of Parliament, power was granted to the Royal College of Surgeons to examine candidates for the diploma of Licentiate in Dental Surgery, and to grant that degree ; that in the International Medical Congress there is a section for diseases of the teeth or dental surgery, and that Edwin Saunders, a celebrated dental surgeon, is president of the section ; that the Congress has been addressed by various eminent dentists from different parts of the world, and all the proceedings are published in the *British Medical Journal.*

This affiant further states, that, being a regularly-bred and duly licensed dental surgeon, practicing his profession as such, the performance of jury duty would work material injury to his interests and injury to many of his patients, and to some of them probably serious injury ; that he is advised by counsel for the Washington Dental Society, of which he is a member, after mature deliberation by such counsel, that he is exempt under the law from jury duty. That he is advised by said counsel to assert his privilege by a challenge as well as by a motion, supported by affidavit, as herein done. He appends hereto the letter of said counsel, and the other papers herewith exhibited and filed. That he is informed and believes that this court, Justice Olin presiding, has held that a dental surgeon is exempt by the law from jury duty, the occasion being the summoning of ———, D. D. S,. to do jury duty.

This affiant further states that he tenders himself ready to verify all the matters herein stated or in the foregoing motion stated, and that the notice and summons exhibited to the court herewith and hereto appended is the notice and summons served on him as in the motion stated.

———.

Subscribed and sworn to before me, this 13th day of February, 1883.

R. J. MEIGS, *Clerk*,

By J. J. CAMP, *Asst. Clerk.*

---

### ARTICLE II.

### *The Hygienic Relations of Artificial Dentures.*

EDITOR OF THE AMERICAN JOURNAL OF DENTAL SCIENCE.

